# Factors Contributing to Malnutrition in Parkinson's Disease Patients With Freezing of Gait

**DOI:** 10.3389/fneur.2022.816315

**Published:** 2022-03-11

**Authors:** Li-Li Zhang, Liang Zhang, Jingde Dong, Ying Zhao, Xiao-Ping Wang

**Affiliations:** ^1^Shanghai General Hospital of Nanjing Medical University, Shanghai, China; ^2^Department of Geriatrics, Affiliated Brain Hospital of Nanjing Medical University, Nanjing, China; ^3^Department of Neurology, Affiliated Hospital of Jiangnan University, Wuxi, China

**Keywords:** Parkinson's disease, freezing of gait, motor symptoms, non-motor symptoms, malnutrition

## Abstract

**Background and Purpose:**

Little is known about the nutritional status and clinical characteristics of patients with Parkinson's disease with freezing of gait (PDFOG). The purpose of this study was to describe the relationship between nutritional status and characteristics of patients with PDFOG.

**Methods:**

In this cross-sectional study, 178 PDFOG patients were recruited and classified as nutritionally normal or at risk of malnutrition/already malnourished based on their Mini Nutritional Assessment (MNA) scores. Each participant underwent a structured questionnaire, physical examination and routine serum biochemical tests.

**Results:**

We found that 44.4 and 37.1% of PDFOG patients were malnourished [mini nutritional assessment (MNA) score <17] and at risk of malnutrition (17 ≤ MNA score ≤ 23.5), respectively. Compared to patients with normal nutrition, PDFOG patients with malnutrition and at risk of malnutrition had longer duration of Parkinson's disease (PD) and freezing of gait (FOG), more levodopa equivalent daily doses (LEDD), lower body mass index (BMI), more motor symptoms according to the Unified PD Rating Scale-III (UPDRS-III) and non-motor symptoms according to the PD Non-motor Symptoms Questionnaire (PD-NMS) (*P* < 0.05). Uric acid, albumin, prealbumin, and total cholesterol (TC) differed between the two groups (*P* < 0.05). High Hoehn and Yahr (H-Y) stage, high Freezing of Gait Questionnaire (FOGQ) scores, low TC and low uric acid were risk factors for malnutrition in patients with PDFOG.

**Conclusion:**

Our results showed disease severity, motor symptoms, TC levels and uric acid levels were all associated with nutritional status in patients with PDFOG. This study suggest early discovery of the nutritional status of PDFOG patients is important.

## Introduction

Parkinson's disease (PD) is one of the most common neurodegenerative disorders associated with the progressive degeneration of dopaminergic neurons in substantia nigra and the formation of Lewy bodies. PD is characterized by both motor and non-motor symptoms (1). The nutritional status of patients with PD is closely related to their quality of life (2). Our team and previous studies have confirmed that patients with PD are at high risk for malnutrition (3, 4). Several studies have demonstrated that the incidence of PD can be reduced by changing dietary outcomes, such as reducing milk and increasing intake of vegetables, fruits and fish, having a high diet quality or a healthy dietary pattern (5, 6). Duration of PD, motor and non-motor PD symptoms are closely related to nutritional status (7). Patients with PD are less likely to adhere to a Mediterranean-style diet compared to controls (8).

Freezing of gait (FOG) is a complex condition. It is characterized by “an inability to produce effective footsteps, and these usually occur during gait initiation or turning while walking” (9). More than half of patients with PD have experienced FOG during the course of their disease (10). FOG is not only a major risk factor for falls in patients with PD, it is also a source of disability for patients with PD. At present, the treatment of FOG mainly includes drug treatment, surgical treatment, physical therapy and occupational therapy (9, 11). The research on the treatment of PDFOG is in progress.

Nutritional risk may play an important role in PD. However, little is known about the nutritional status and clinical characteristics of patients with Parkinson's disease with freezing of gait (PDFOG). The aim of this study was to describe the relationship between nutritional status and characteristics of patients with PDFOG.

## Patients and Methods

### Patients

Consecutive series of patients diagnosed with PDFOG from January 2015 to December 2020 at the Department of Neurology and Geriatrics, Affiliated Brain Hospital of Nanjing Medical University were recruited to this study. Diagnostic criteria for PDFOG:1 PD diagnostic criteria meeting the British Parkinson's Disease Association Brain Bank Clinical Diagnostic Criteria (12). In total 2 Diagnostic criteria for FOG: According to Factor and Giladi, using FOG-Q item 3 (score >0), it is defined as a sudden gait disturbance of short duration and unpredictability (9, 13, 14). The feet seem to stick to the ground and cannot move. Symptoms are exacerbated when starting, turning, or passing through narrow areas such as passages and doors (3). All included PD patients had FOG during the course of the disease. The exclusion criteria were as follows (1). Patients with other medical conditions such as: autoimmune disorder, history of hematopoietic stem cell transplant, lung disease (e. g. asthma, COPD), solid organ cancer, heart disease (e.g. coronary artery disease, heart failure), hematologic cancer, vascular disease (e.g. peripheral vascular disease), kidney disease, psychiatric disorders (e.g. depression, anxiety, schizophrenia, mania), other neurological diseases (e.g. Alzheimer's disease, Multiple system atrophy, Progressive supranuclear ophthalmoplegia). In total 2 Patients were unable to complete all examinations. Diagnosis and assessment of the scales were performed by experienced neurologists.

This study was approved by the Ethics Committee of Affiliated Brain Hospital of Nanjing Medical University, and the approval numbers were “2015-KY057-01”. This study was performed in accordance with the Declaration of Helsinki. We obtained written informed consent from all patients before collecting the clinical information.

### Neuropsychological Assessment

The Mini Nutritional Assessment (MNA) was used to classify patients as having normal nutritional status, at risk of malnutrition, or already malnourished (MNA score 24–30, normal nutritional status; MNA score 17–23.5, at risk of malnutrition; MNA score <17, already malnourished) (15, 16). Patients' age, sex, duration of PD disease, duration of FOG disease, weight, levodopa equivalent daily dose (LEDD) (17), and body mass index (BMI) were recorded using standard forms. BMI was a commonly used international standard for measuring obesity levels and health status. It wascalculated as BMI = weight in kilograms ÷ height in square meters. LEDD was calculated based on previously published recommendations. The Unified PD Rating Scale III (UPDRS-III) and Hoehn and Yahr (H-Y) stages were used to assess motor symptoms (18). The Non-Motor Symptom Questionnaire (NMSQ) was used to record non-motor symptoms (19). The Montreal Cognitive Assessment (MOCA) was used to measure general cognitive ability. The Hamilton Anxiety Inventory (HAMA) and Hamilton Depression Inventory (HAMD) were used to assess mood. Five milliliters of venous blood were collected at 6:00 am after admission and blood biochemical parameters [uric acid, albumin, prealbumin, total cholesterol (TC), high-density lipoprotein blood (HDLC), low-lipoprotein blood (LDLC), and serum triglycerides (TG) were measured. (Biochemistry analyzer, Olympus).

### Statistical Analysis

IBM SPSS software version 25.0 was used to evaluate all data in this study.

Numerical data were expressed as mean ± standard deviation (SD). Categorical variables were described by frequencies and percentages. Independent samples *t*-tests and Chi-Square tests were used to compare baseline sample characteristics, neuropsychological assessments, and blood indicators. We used forward logistic regression for multivariate analysis, with nutrition as the dependent variable and significant disease characteristics [PD duration, FOG duration, LEDD, UPDRS-III, H-Y stage, PD-NMS, uric acid, albumin, pre-albumin, TC] as independent covariates, for exploring potential clinical factors that may be related to nutrition between at risk of malnutrition, malnourished groups and the nutrition normal group. *P* < 0.05 was considered statistically significant.

## Results

### Demographic Characteristics and Clinical Symptoms Results

The demographic and clinical characteristics of all 178 patients with PDFOG are summarized in Table 1. The mean duration of PD disease was (14.57 ± 7.80) years, with a range of 0.7–28 years. According to the MNA, we found that 44.4 and 37.1% of PDFOG patients were malnourished [mini nutritional assessment (MNA) score <17] and at risk of malnutrition (17 ≤ MNA score ≤ 23.5), respectively.

**Table 1:** Demographical characteristics and clinical symptoms of PDFOG patients.

**Variables**	**Total (*N* = 178)**	**Range**
Sex (male %)	103 (57.9%)	-
Age, mean ± SD	68.72 ± 14.17	46–92
Duration of PD disease, years	14.57 ± 7.80	0.7–28
Duration of FOG, years	5.72 ± 2.97	0.5–25
LEDD, mg, mean ± SD	538.61 ± 108.78	0–850
Body weight (Kg)	62.61 ± 12.86	43–81
BMI, mean ± SD	19.46 ± 3.02	15.0–30.0
Normal nutrition status (*N*, %)	33 (18.5%)	-
At risk of malnutrition (*N*, %)	66 (37.1%)	-
Malnourished (*N*, %)	79 (44.4%)	-
UPDRS-III, mean ± SD	40.49 ± 12.63	9–78
H-Y stage, mean ± SD	3.24 ± 0.85	2–5
FOGQ, mean ± SD	13.06 ± 4.29	3–24
MOCA, mean ± SD	22.85 ± 3.89	0–30
PD-NMS, mean ± SD	16.45 ± 5.25	0–30
HAMA	13.22 ± 6.06	0–36
HAMD	18.79 ± 8.23	0–49

*PD, Parkinson's disease; FOG, Freezing of Gait; LEDD, Levodopa equivalent daily dose; BMI, Body mass index; UPDRS-III, Unified PD Rating Scale; H-Y stage, Hoehn and Yahr stage; FOGQ, Freezing of Gait Questionnaire; MOCA, Montreal Cognitive Assessment; NMS, Non-Motor Symptoms Questionnaire; HAMA, Hamilton Anxiety Rating Scale; HAMD, Hamilton Depression Rating Scale*.

We compared general clinical data between PDFOG patients at risk of malnutrition, malnourished patients, and nutritionally normal PDFOG patients. No significant differences were found in age, sex, MOCA scores, HAMD scores or HAMA scores. Duration of PD disease, duration of FOG, LEDD, weight, BMI, UPDRS-III scores, H-Y stage, FOGQ scores and PD-NMS scores, were significantly different between the two groups. PDFOG patients at risk of malnutrition and malnourished had longer duration of PD (*P* = 0.00) and FOG (*P* = 0.01), more LEDD (*P* = 0.03), lower weight (*P* = 0.00) and BMI *(P* = 0.00), and worse motor symptoms (*P* = 0.00), FOG (*P* = 0.00), and non-motor symptoms (*P* = 0.00) (Table 2).

**Table 2:** The general clinical data of PDFOG patients at risk of malnutrition, malnourished, and patients with normal nutrition.

**Variables**	**At risk of malnutrition and malnourished (*N* = 145)**	**Normal nutrition** **(*N* = 33)**	***t***	*P* ^*^
Sex (male %)	85 (58.6%)	15 (45.5%)	1.89	0.18^a^
Age, mean ± SD	69.34 ± 14.02	66.00 ± 14.74	−1.22	0.75^b^
PD duration of disease, years	16.32 ± 6.97	6.87 ± 6.54	−7.40	**0.00** ^b^
FOG duration of disease, years	5.98 ± 2.72	4.58 ± 3.73	−2.47	**0.01** ^b^
LEDD, mg, mean ± SD	547.13 ± 104.37	501.16 ± 121.09	−2.21	**0.03** ^b^
Body weight (Kg)	61.80 ± 10.63	66.15 ± 15.95	1.92	**0.00** ^b^
BMI, mean ± SD	18.96 ± 2.41	21.65 ± 4.26	4.91	**0.00** ^b^
UPDRS-III, mean ± SD	43.11 ± 11.28	29.00 ± 11.96	−6.18	**0.00** ^b^
H-Y stage, mean ± SD	3.41 ± 0.74	2.48 ± 0.89	−5.52	**0.00** ^b^
FOGQ, mean ± SD	14.36 ± 3.33	7.33 ± 3.24	−11.2	**0.00** ^b^
MOCA, mean ± SD	22.95 ± 3.77	22.45 ± 4.41	−5.99	0.55^b^
PD-NMS, mean ± SD	17.07 ± 4.79	13.73 ± 6.29	−3.41	**0.00** ^b^
HAMD	18.33 ± 8.25	20.82 ± 7.96	1.61	0.11^b^
HAMA	13.45 ± 6.22	12.24 ± 5.23	−1.15	0.25^b^

* *Patients at risk of malnutrition and malnourished were compared with patients with normal nutrition.*

a *Chi-square test.*

b *T-test. Bold values mean emphasize results P < 0.05, which we set as having statistical differences. PD, Parkinson's disease; FOG, Freezing of Gait; LEDD, Levodopa equivalent daily dose; BMI, Body mass index; UPDRS-III, Unified PD Rating Scale; H-Y stage, Hoehn and Yahr stage; FOGQ, Freezing of Gait Questionnaire; MOCA, Montreal Cognitive Assessment; NMS, Non-Motor Symptoms Questionnaire; HAMA, Hamilton Anxiety Rating Scale; HAMD, Hamilton Depression Rating Scale*.

### Biochemical and Blood Indices of Patients With PDFOG Based on Nutritional Status

Biochemical and blood indices were also compared in PD patients at risk of malnutrition, malnourished group and nutritionally normal group. The results showed that PD patients at risk of malnutrition and malnourished group had lower uric acid, albumin, prealbumin, and total cholesterol (TC) (*P* < 0.05) (Table 3).

**Table 3:** Biochemical and blood indicators of PDFOG patients based on nutritional status.

**Variables**	**At risk of malnutrition and malnourished (*N* = 145)**	**Normal nutrition (*N* = 33)**	***t***	P^*^
Uric acid	192.08 ± 80.54	354.35 ± 98.92	9.99	**0.00^a^**
Albumin	32.57 ± 5.75	38.33 ± 7.54	4.88	**0.00^a^**
Pre-albumin	206.83 ± 43.06	285.88 ± 53.09	7.97	**0.00^a^**
TC	2.96 ± 0.59	4.27 ± 0.92	10.25	**0.00^a^**
HDLC	1.18 ± 0.15	1.21 ± 0.13	0.89	0.37^a^
LDLC	1.81 ± 0.18	1.85 ± 0.21	0.95	0.35^a^
Triglyceride	1.55 ± 0.39	1.51 ± 0.38	−0.53	0.59^a^

* *Patients at risk of malnutrition and malnourished were compared with patients with normal nutrition.*

a *T-test. Bold values mean emphasize results P < 0.05, which we set as having statistical differences. TC, total cholesterol; HDLC, high density lipoprotein; LDLC, low high density lipoprotein*.

### Risk Factors for Malnutrition in PDFOG Patients

The nutritional status was used as the dependent variable. Variables that were significantly different between the two groups with different nutritional status: duration of PD disease, duration of FOG, LEDD, UPDRS-III score, H-Y stage, FOGQ score, PD-NMS score; uric acid, albumin, and pre-albumin, were included in the logistic regression analysis to assess the relationship between the above factors and malnutrition in PDFOG patients. The results showed that higher H-Y staging (*P* = 0.009), more FOGQ scores (*P* = 0.009), lower TC levels (*P* = 0.020), and lower Uric acid were risk factors for malnutrition in PDFOG patients (Table 4).

**Table 4:** Multivariate unconditional logistic regression analysis was used on risk factors for malnutrition.

**Variables**	**OR**	**95%Cl**	***P* ^a^**
LEDD	0.980	0.957–1.004	0.095
H-Y stage	17.369	2.017–149.599	**0.009**
FOGQ	6.923	1.614–29.702	**0.009**
Albumin	1.312	0.957–1.80.	0.092
Pre-albumin	0.975	0.950–1.002	0.067
TC	0.041	0.003–0.602	**0.020**
Uric acid	0.958	0.924–0.993	**0.020**

a *The P-value is calculated from a forward binary logistic regression analysis. OR, odds ratio; CI, confidence interval; LEDD, Levodopa equivalent daily dose; FOGQ, Freezing of Gait Questionnaire; TC, total cholesterol. Bold values mean emphasize results P < 0.05*.

## Discussion

Our study explored the nutritional status of PDFOG patients and possible associated factors. According to MNA, 44.4% of PDFOG patients were malnourished and 37.1% of PDFOG patients were at risk of malnutrition. Our previous study showed that 39.2% of PD patients were malnourished and 30.0% of PD patients were at risk for malnutrition (3). A systematic review showed that the prevalence of malnutrition in PD patients was 0–24%, and 3–60% of PD patients were at risk of malnutrition (20). It suggests that PD patients with FOG may have a higher risk of malnutrition than PD patients. FOG reduced independently the quality of life (21). The nutritional status of patients with Parkinson's disease is also closely related to quality of life (2). Early identification of risk factors for malnutrition in patients with PDFOG, timely and effective interventions are important for the quality of life of patients with PDFOG.

We analyzed the relationship between disease duration, BMI, LEDD, motor symptoms, non-motor symptoms, and nutritional status in PDFOG patients. Patients with PDFOG who were malnourished and at risk of malnutrition had a longer disease duration, more severe H-Y staging, lower BMI, and more LEDD than PDFOG patients with normal nutritional status.

Many studies have confirmed that the nutritional status of patients with Parkinson's disease becomes worse with the duration of the disease. Both the duration and severity of the disease are also associated with FOG. A meta-analysis showed that the prevalence of FOG in PD patients was 39.9% (22). Among PD patients, the prevalence of FOG was highest in patients with disease duration longer than 9 years (64.6%), and patients with PD of ≤ 5 years (37.9%) (23). In total 28.4% of PDFOG patients had HY stage ≤ 2.5, but 68.4% of PDFOG patients had H-Y stage ≥2.5 (22).

The results of this study suggested that the nutritional status of PDFOG patients were associated with motor symptoms: higher UPDRS-III scores and FOGQ scores. Previous studies have also shown that malnutrition in PD patients is associated with UPDRS-III. A meta-analysis showed that motor symptoms (UPDRS-III scores) affect BMI in PD patients (24). High UPDRS-III scores may indicate more severe symptoms of stiffness and sluggishness, leading to increased energy expenditure in patients. The more severe the symptoms, the greater the increase in energy expenditure. It is also still shown that patients' mobility retardation affects patients' access to food, but at the same time, energy expenditure decreases due to reduced exercise. Thus, the conclusions regarding the effect of various motor symptoms on the nutritional status of PD patients have been inconsistent across studies.

The PD-NMS scale can quantify non-exercise symptoms well. The results of this study showed that patients with PDFOG at risk of malnutrition and the malnourished group had higher scores of non-motor symptoms than the nutritionally normal group. There are many studies on the influence of non-motor symptoms affecting nutritional status in PD. Body weight is significantly associated with cognitive performance. Dementia or visual hallucinations can and do produce weight loss. This may be related to decreased appetite or negative energy balance due to neuroendocrine dysfunction (4). A cohort study involving 398 patients with PD found a strong relationship between weight loss and nigrostriatal deficits (20). The gut may be involved in the pathogenesis of Parkinson's disease, influencing the entire course of the disease. PD-associated gastrointestinal dysfunctions, including oral disorder, dysphagia, excess salivary acid, dysphagia, gastroparesis, and defecation dysfunction all may occur. These dysfunctions put patients at risk for malnutrition (25).

Nutritional status was also associated with higher LEDD and indices of disease progression (severity of motor symptoms and dysphagia) (26). Levodopa may cause dyskinesia, increase energy expenditure, and impair masticatory function. Long-term use has direct effects on fat metabolism, including skeletal muscle glucose uptake and prolactin secretion associated with eating behavior, leading to nausea and anorexia (27). LEDD was associated with worse nutritional status and risk of malnutrition.

Our study showed that uric acid was strongly associated with malnutrition. Serum uric acid levels were associated with non-motor symptoms such as dysphagia, anxiety, depression, apathy and cognitive dysfunction, as well as whole brain gray matter volume (28). Uric acid was a protective factor for PD patients and has some predictive value for PD (29). Uric acid is a natural antioxidant that protects dopaminergic neurons. Low levels of uric acid lose its protective effect.

In recent years, several studies have confirmed lipid levels and neuronal function. Stable lipid levels facilitate normal neuronal function (30, 31). Circulating total cholesterol may play an important role in the development and progression of PD. This may partly explain its inverse relationship with Parkinson's disease (32). Lipid signaling is involved in oxidative stress responses, lysosomal function, endoplasmic reticulum stress responses, and immune response activation (33). Cholesterol may regulate presynaptic dopamine homeostasis *in vitro*. TC may also image the non-motor symptoms of PD (34), and may be involved in α-synuclein aggregation and accumulation (35). High dietary intake of high cholesterol may have a protective effect on PD pathogenesis. Although the underlying mechanisms of the association between cholesterol and the nutritional status of PDFOG are unclear, more studies are needed in the future to investigate the role of cholesterol in PD and PDFOG disease (36).

As we have previously reported, pharmacological treatments for FOG are limited and clinicians are seeking multifaceted therapeutic approaches (11). Nutritional status is associated with quality of life in patients with PDFOG. Improving the quality of life of patients by improving their nutritional status deserves further exploration.

It is strongly recommended to evaluate the nutritional status of patients with PD to provide information to determine the necessary nutritional interventions. Clinicians should monitor body weight and nutritional status from the beginning of diagnosis and throughout the follow-up period.

The limitation of our study is that this is a single-center cross-sectional study, and it may be difficult to establish a clear causal relationship. We are conducting a five-year multicenter prospective study to explore the nutritional status and related predictive factors of PD patients and PDFOG patients.

## Conclusions

Freezing of gait is a common symptom in patients with PD and affects the quality of life. PDFOG patients are at higher risk of malnutrition. High H-Y stages, High FOGQ score, low levels of TC and Uric were associated with nutritional status among PDFOG patients. Early assessment and intervention in PDFOG patients may improve their treatment and management outcomes.

## Data Availability Statement

The original contributions presented in the study are included in the article/supplementary material, further inquiries can be directed to the corresponding author/s.

## Ethics Statement

The studies involving human participants were reviewed and approved by the Ethics Committee of Affiliated Brain Hospital of Nanjing Medical University (approval number 2015-KY057-01). The patients/participants provided their written informed consent to participate in this study. Written informed consent was obtained from the individual(s) for the publication of any potentially identifiable images or data included in this article.

## Author Contributions

X-PW and L-LZ were involved in the conception and design of the study, acquisition of primary data, analysis of data, drafting the article, and revising the manuscript. JD, L-LZ, and YZ were involved in the study concept and design and data collection and analysis. All authors contributed to this paper and approved the submitted version.

## Funding

This work was supported by the Science and Technology Development Fund of Nanjing University (NMUB2019097).

## Conflict of Interest

The authors declare that the research was conducted in the absence of any commercial or financial relationships that could be construed as a potential conflict of interest.

## Publisher's Note

All claims expressed in this article are solely those of the authors and do not necessarily represent those of their affiliated organizations, or those of the publisher, the editors and the reviewers. Any product that may be evaluated in this article, or claim that may be made by its manufacturer, is not guaranteed or endorsed by the publisher.
